# A cell free biomembrane platform for multimodal study of influenza virus hemagglutinin and for evaluation of entry-inhibitors against hemagglutinin

**DOI:** 10.3389/fmolb.2022.1017338

**Published:** 2022-10-13

**Authors:** Arpita Roy, Sylvester Byrne, Nirod Kumar Sarangi, Paul V. Murphy, Tia E. Keyes

**Affiliations:** ^1^ School of Chemical Sciences and National Centre for Sensor Research, Dublin City University, Dublin, Ireland; ^2^ School of Biological and Chemical Sciences, University of Galway, Galway, Ireland

**Keywords:** hemagglutinin (HA), influenza entry inhibitor, drug discovery, microfluidic, fluorescence correlation spectroscopy (FCS), electrochemical impedance spectroscopy (EIS)

## Abstract

Seasonal periodic pandemics and epidemics caused by Influenza A viruses (IAVs) are associated with high morbidity and mortality worldwide. They are frequent and unpredictable in severity so there is a need for biophysical platforms that can be used to provide both mechanistic insights into influenza virulence and its potential treatment by anti-IAV agents. Host membrane viral association through the glycoprotein hemagglutinin (HA) of IAVs is one of the primary steps in infection. HA is thus a potential target for drug discovery and development against influenza. Deconvolution of the multivalent interactions of HA at the interfaces of the host cell membrane can help unravel therapeutic targets. In this contribution, we reported the effect of a multivalent HA glycoprotein association on various glycosphingolipid receptors (GD1a, GM3, GM1) doped asymmetrically into an artificial host membrane spanned across an aqueous filled microcavity array. The extent of HA association and its impact on membrane resistance, capacitance, and diffusivity was measured using highly sensitive electrochemical impedance spectroscopy (EIS) and fluorescence lifetime correlation spectroscopy (FLCS). Furthermore, we investigated the inhibition of the influenza HA glycoprotein association with the host mimetic surface by natural and synthetic sialic acid-based inhibitors (sialic acid, Siaα2,3-GalOMe, FB127, 3-sialyl lactose) using electrochemical impedance spectroscopy and observe that while all inhibit, they do not prevent host binding. Overall, the work demonstrates the platform provides a label-free screening platform for the biophysical evaluation of new inhibitors in the development of potential therapeutics for IAV infection prevention and treatment.

## Introduction

We have seen the devastating impact of viral infection when it reaches pandemic level over the past 3 years. While not the only threat, influenza has the demonstrated capacity to cause pandemic level infections and very high mortality. Influenza even at sub-pandemic levels, results in millions of viral infections and significant mortality each year. Furthermore, there has been concern that an influenza variant could emerge that would give rise to a pandemic as experienced in recent years or in 1918 (Molinari et al., 2007; Iuliano et al., 2017). Virus attachment to the host plasma membrane is the first critical step in infection and is a key factor contributing toward disease virulence (Li et al., 2021). Enveloped viruses feature a membrane that surrounds the nucleocapsid containing the genome. The envelope contains viral glycoproteins that mediate interaction with receptors in the plasma membrane of the host cell (Sonnino et al., 2006; Du et al., 2019). Membrane fusion releases the viral RNA genome into the cytoplasm of the host cell where the virus replicates. For example, in the case of influenza A and B viruses, the hemagglutinin (HA) viral glycoprotein is involved in the membrane binding process. HA comprises of three monomers that consists of the membrane-distal globular head domain (HA1) and the membrane-proximal domain (HA2). The HA1 subunits contain the receptor-binding domain (RBD) that enables viral attachment to the cell surface via association with sialic acid (SA) receptors at the host membrane, and the HA2 subunit mediates membrane fusion (Mammenet al., 1998; Guo et al., 2017). The RBD recognition of SA is the first step in infection and is conserved across influenza viruses. It is thus, a rational target for therapeutic intervention. Influenza A viruses have different subtypes depending on the antigenic natures of the two glycoproteins neuraminidase (NA) and hemagglutinin (HA) (Medina and García-sastre, 2011).

Importantly, multivalency pervades the mechanisms of viral-receptor interaction pathways, where the additive thermodynamic impact of multiple weak interactions promotes avidity and selectivity in receptor binding in host-virus interactions (Mammenet al., 1998). This multivalency influences the motion of virus particles at the host cell surface (Bally et al., 2021), as well as aiding in membrane reorganization. However, multivalent interactions make the overall viral-entry process so complex that it is extremely difficult to therapeutically inhibit the viral-cell entry.

Thus, it is important to replicate multivalency in virus-receptor interactions in model systems in order to disentangle the complex mechanisms behind receptor-mediated viral uptake and its impact on the host cell membrane. Such insight can contribute to a better understanding of infection and moreover, can facilitate in the design of perspective inhibitors for the blocking of viral entry pathways.

The interaction between lipids and proteins within the host membrane also plays a crucial role in viral infection (Carton et al., 2010; Siontorou et al., 2017). According to the raft hypothesis, the plasma membrane contains coexisting ordered and disordered domains that influence HA-glycan binding (Nicolau et al., 2006; Sezgin et al., 2017; Goronzy et al., 2018). Rafts are believed to be fluctuating patches of membrane primarily composed of unsaturated phospholipids, sphingolipids, cholesterol with diameters ranging from nm to micron (Gaus et al., 2003; Kusumi and Suzuki, 2005; Parton et al., 2013; Rosetti et al., 2017). Rafts within the natural membrane are believed to bear analogy to the domains of “liquid ordered” (L_o_) and “liquid-disordered” (L_d_) phases that are observed in biophysical mixtures of lipids. The L_o_ phase has restricted mobility with densely packed saturated lipid chains, and it is rich in cholesterol and sphingolipid. The L_d_ phase is more fluid compared to L_o_ comprising unsaturated lipids with lower cholesterol content (Veit and Thaa, 2011; Parton et al., 2013).

While it has been speculated that lipid rafts play a crucial role in influenza infection, evidence for this role is still emerging. It has been reported from liposome-based studies that HA partitions selectively into liquid ordered (L_o_) domains, and subsequently promotes clustering and budding of virus (Scheiffele et al., 1999; Veit and Thaa, 2011). However, the exact nature of raft association with influenza hemagglutinin needs further investigations due to complexity of *in vivo* measurements (Lingwood and Simons, 2012; Parton et al., 2013). A number of fluorescence resonance energy transfer (FRET)-based investigations have been carried out on living cells which suggested the association of full-length HA with raft markers in the plasma membrane (Takeda et al., 2003; Engel et al., 2010; Verma et al., 2018; Ripa et al., 2021).

Biophysical experimental platforms that have the dynamic properties of the cellular plasma membrane and that can serve as intermediate fluidic devices between the existing solid supported bilayer platforms and complex cellular assays can provide valuable insight into viral infection. There are numerous reports of such model platforms that simplified various complex biochemical processes (Rakowska et al., 2013; Jiang et al., 2014; Orosz et al., 2017; Neumann et al., 2018; Fonseka et al., 2019; Cavassin et al., 2020; Tang et al., 2021; Fonseka et al., 2022). In our previous study, we have investigated the relative affinity of the hemagglutinin ectodomain, the HA1 subunit, towards different GSLs (Berselli et al., 2020). However, the role of different GSLs, host cell membrane composition in full length multivalent influenza glycoprotein HA association as well as its inhibition by potential entry inhibitor drugs or inhibitors has not been studied at a biophysical model yet. This present study focuses on the trimeric influenza HA which is the native form of this glycoprotein (Jegaskanda et al., 2014). We examine the association of this protein at microcavity supported lipid bilayers and the role of host receptor density on virus binding and how this influences the model membrane. This was accomplished by, investigating the effect of HA binding on membrane diffusion properties as well as changes in membrane electrical properties with two different (1 mol% and 5 mol%) receptor densities at host membrane by employing fluorescence correlation spectroscopy, FCS, and electrochemical impedance spectroscopy, EIS studies respectively.

The search for potent therapeutic agents for influenza infection treatment is ongoing. Zanamivir and oseltamivir inhibitors have been developed as neuraminidase inhibitors (Gubareva et al., 2000). *In vitro* investigations have revealed that neuraminidase-resistant strains of influenza virus may evolve quickly in the presence of inhibitors due to small modifications through amino acid substitution in the viral hemagglutinin (HA) protein (Mckimm-breschkin, 2000; Smee et al., 2001). Mutation bears less influence on the structure of the specific oligosaccharides that are included at the glycosylation sites (Keefe et al., 2003). Therefore, the oligosaccharides of the IAV glycoprotein HA may be a useful target against which potential therapeutics might be developed for a wide spectrum of influenza virus strains. Although, there are only few effective entry inhibitor drugs against influenza despite extensive research into effective anti-influenza drug that can inhibit viral entry, (Chang et al., 2021), rapid screening is a key to making progress. Cell free platforms that can be used for rapid screening for viral inhibition have potential value in high throughput studies in the search for effective anti-influenza drugs. Herein, we have performed label-free detection technique EIS (Shkirskiy et al., 2020; Tang et al., 2021) to investigate the effectivity of sialic acid (SA), sialyl lactose (3′SL) and synthetic inhibitors against influenza viral glycoprotein HA entry into the host cell membrane. Our EIS study also reveal that the SA as well as the synthesized inhibitors interact directly with the viral HA protein.

## Experimental section

### Materials

High purity (>99%) 1,2-Dioleoyl-*sn*-glycero-3-phosphocholine (DOPC), porcine brain N-(octadecanoyl)-sphing-4-enine-1-phosphocholine (SM), cholesterol, GD1a, GM3, GM1 were purchased from Avanti Polar Lipids (Alabaster, Alabama) and used as received. 1,2-Dioleoyl-*sn*-glycero-3-phosphoethanolamine-labeled Atto655 (DOPEA655), ATTO532 NHS-ester were brought from ATTO-Tec GmbH. Phosphate-buffered saline (PBS) tablets were purchased from Sigma-Aldrich (Wicklow, Ireland). Influenza A H_3_N_2_ (A/Aichi/2/1968) Hemagglutinin/HA Protein (ECD, His Tag) was purchased from Stratech Scientific Ltd. Aqueous solutions were prepared using Milli-Q water (Millipore, Bedford, MA). Polydimethylsiloxane silicon (PDMS) elastomer was purchased from Dow Corning (Wiesbaden, Germany) and mixed following the supplier’s instructions. Silicon wafers coated with a 100 nm layer of gold on a 50 Å layer of titanium were obtained from AMS Biotechnology. The monodisperse polystyrene latex sphere with a diameter of 1 μm were obtained from Bangs Laboratories. The commercial cyanide-free gold-plating solution (TG-25 RTU) was obtained from Technic. All other HPLC-grade reagents were obtained from Sigma-Aldrich and used as received. The synthesis of FB127 (Woods et al., 2017) and the disaccharide Siaα2,3GalOMe (Dhakal et al., 2016) are given in the supplementary information section (Supplementary Schemes S1,S2).

### Fluorescent labelling of HA

For FCS studies, labeling of influenza A, H_3_N_2_, viral glycoprotein HA with ATTO532 NHS-ester was accomplished at basic pH by reaction of terminal amines in HA with the succinimidyl ester moiety of ATTO532 dye. To avoid ester hydrolysis and maintain the dye in its reactive form ATTO532 NHS-ester was first diluted in anhydrous DMSO (HPLC grade).

The HA solution was made up in PBS buffer at pH 8.3 such that the final concentration was 0.2 mg/ml. ATTO532 NHS-ester in a molar ratio of 5:1 (dye to protein) was added to the HA solution and reaction proceeded for 1 h in absence of light with gentle agitation of the reaction mixture. The labelled protein was purified by dialysis using a centrifugal filter membrane (AMICON 10 kDa) carried out at 10,000 RPM for 15 min. This removed unreacted dye. This procedure was performed 6 times by adding 1 ml PBS buffer at pH 7.4 each time. UV-VIS spectroscopic measurement (Supplementary Figure S1) and FLCS of ATTO532 labeled HA was carried out to confirm HA labelling. The lateral diffusion coefficient of ATTO532 labeled HA in PBS at pH 7.4 was determined by fitting the autocorrelation functions (ACFs) in 3D diffusion model.

### Fabrication of PDMS and gold microcavity arrays

Microcavity arrays were constructed in PDMS for the FLCS experiments or in gold on silicon wafer for EIS studies by polystyrene sphere templating as per earlier reports (Gimenez et al., 2020; Robinson et al., 2020). In brief, for the fabrication of gold arrays, the PS microsphere of 1 μm diameter were drop cast into the gold-coated silicon wafers by utilizing the gravity assisted technique to form a highly ordered array. Then, electrochemical deposition was used to deposit gold into the electrode around the array to 50% of the sphere height (Maher et al., 2016; Basit et al., 2017). Finally, the top interpore surface of the gold array electrodes were selectively modified with self-assembled monolayer (SAM) of 1 mM 6-mercapto-1-hexanol (MH). This SAM is required to promote bilayer stability. Finally, the PS sphere template was removed by washing with THF and ethanol to yield top surface SAM modified gold arrays. The electrodes are sonicated in buffer and kept in contact with working buffer until use.

To fabricate substrates for microscopy, optically transparent PDMS microcavity arrays were prepared by drop casting 4.6 μm PS microsphere onto a mica sheet. PDMS was poured across mica sheet and curing was completed at 90 °C for approximately 1 h until the PDMS becomes hard. The resulting PDMS substrate was peeled off the mica and the resulting microcavity array was sonicated in tetrahydrofuran (THF) for about 15 min to remove the PS sphere templates. Prior to filling the cavities with PBS buffer pH 7.4, the substrate was treated by oxygen plasma for 5 min to render it hydrophilic and then sonicated in PBS buffer for 15 min.

For both gold and polymer arrays the same bilayer deposition procedure was applied; lipid monolayer transfer was accomplished using Langmuir-Blodgett deposition. Usually, 50 μl of lipids (1 mg/ml in chloroform) was added dropwise onto the subphase (Milli Q water, 18.2 MΩ cm) at room temperature (20 ± 1°C), and chloroform was then evaporated for 7 min. The monolayers were subjected to four cycles of compression/decompression at a barrier speed of 20 mm/min not exceeding the final surface pressure (Π) of 35 mN m^−1^. Then, the monolayer is compressed up to 32 mN m^−1^ and held for at least 300 s before transfer. Lipid monolayers were transferred onto the hydrophilic gold and PDMS substrates vertically up at a speed of 5 and 10 mm/min respectively. Then, the monolayer formed-substrates were immersed in a 0.25 mg/ml liposome solution, and the fusion was allowed to occur for 1–1.5 h to form bilayers. The substrate was maintained in contact with liposome solution throughout the fusion. The substrate was washed gently with working buffer (PBS pH 7.4) to remove any unfused liposomes and was kept in contact with this buffer while being transferred to the electrochemical chamber. Similarly, for the PDMS substrate, the chamber was washed 3 to 4 times with working buffer (PBS pH 7.4) and kept in contact with this solution until the experiment was completed to prevent the bilayer drying.

### Characterization of gold/PDMS microcavity arrays

The shape and size of the formed microcavity arrays were confirmed by scanning electron microscopy (SEM). SEM images of gold arrays were obtained using a Hitachi S5500 (Supplementary Figure S2). All images were acquired using the secondary electron mode.

### FLCS measurements

FLCS measurements were performed on the labeled HA-ATTO532 and DOPE-ATTO655 to confirm the incorporation of HA into MSLBs and to investigate its effect on the host bilayer membrane. FLCS measurements were performed on a MicroTime 200 lifetime (PicoQuant GmbH, Berlin, Germany) using a water immersion objective (NA 1.2 UPlanSApo 60 × 1.2 CC1.48, Olympus). The detection unit comprises two single photon avalanche diodes from PicoQuant. A labeled lipid membrane marker DOPE-ATTO655 was excited with 640 nm LDH-P-C-640B (PicoQuant), and HA-ATTO532 was excited with a 532 nm PicoTA laser from Toptica (PicoQuant). To exclude scattered or reflected laser light, emitted fluorescence was collected through an HG670lp AHF/Chroma or HQ550lp AHF/Chroma band pass filter for 640 or 532 nm lasers, respectively. A 50 μm pinhole was used to eliminate photons from outside the confocal volume. Before FCLS measurements, backscattered images of the substrate were taken using an OD3 density filter to ensure the optimal positioning of the focus to the center of the microcavity. Then, the bilayer position was determined by z-scanning until the point of maximal fluorescence intensity of DOPE-ATTO655 was found. At this point, the fluctuating fluorescence intensity of the labeled lipid marker or HA-ATTO532 was measured for 30s per cavity with data averaged across 10 to 20 cavities. To assess the diffusion time (ms), the emitted photons were analyzed by a time-correlated single photon counting system (PicoHarp 300 from PicoQuant). The fluorescence fluctuations obtained are then correlated with a normalized autocorrelation function (Eq. 1),

$$G\left(\tau \right)=\frac{\langle \delta F\left(t\right)\delta F\left(t+\tau \right)\rangle }{{\langle F\left(t\right)\rangle }^{2}}$$ (1)

The autocorrelation curves obtained from the fluorescence fluctuations of DOPE-ATTO655 and HA-ATTO532 in bilayer were fitted to a 2-D model (Eq. 2) using the software SymphoTime (SPT64) version 2.4 (PicoQuant).

$$G\left(\tau \right)=\frac{1}{N}{\left[1+{\left(\frac{\tau }{\tau_{D}}\right)}^{\alpha }\right]}^{-1}$$ (2)

Here, α is the anomalous parameter, N is the number of molecules and τ_D_ is the diffusion time of the fluorescent marked molecules in the lipid membrane. The diffusion coefficient is related to the correlation time τ_D_ by the relation D = ω^2^/4τ_D_, where ω is the 1/e^2^ radius of the confocal volume, that is, the waist of the exciting laser beam. ω was measured for each excitation wavelength using a reference solution of free dye for which the diffusion coefficient is known. The ω was determined by calibration using reference dyes; ATTO-655 (AttoTEC, GmbH) for a 640 nm laser and ATTO-532 for a 532 nm laser at 20°C in water.

$$G\left(\tau \right)=\frac{1}{N}{\left[1+{\left(\frac{\tau }{\tau_{D}}\right)}^{\alpha }\right]}^{-1}{\left[1+{\left(\frac{\tau }{\tau_{D}}\right)}^{\alpha }\frac{1}{\kappa^{2}}\right]}^{-1/2}$$ (3)

The lateral diffusion of labelled HA-ATTO532 and the free ATTO532, e.g., unbound dye in PBS buffer, were calculated by fitting the ACFs obtained in for 10 nM solution of each molecule to a 3D model (Eq. 3). Eq. 3 includes a κ term, which defines the shape of the confocal volume.

## Results

### Impact of GSL and multivalent influenza full viral HA protein recognition on electrochemical properties of the MSLB

**SCHEME 1:**
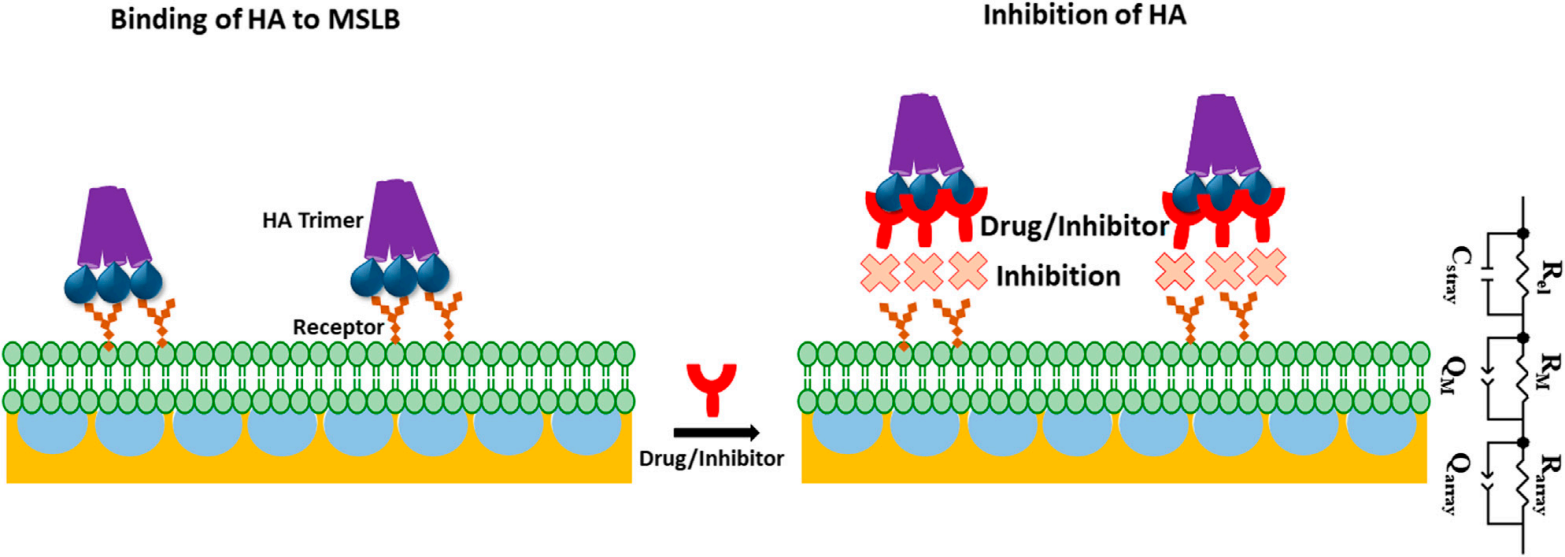
Schematic representation of gold microcavity suspended bilayer array (light green) with embedded receptors (orange) and its interaction with the HA trimeric protein (purple) used in this work. The inhibition of HA incorporation into the bilayer in presence of drug or inhibitor (red) has also been picturized. ECM model is shown that is used to fit EIS data. In the ECM; *R*_el_: electrolyte resistance, *C*_stray_: stray capacitance, *R*_array_ and *R*_M_, represents cavity array and membrane resistance respectively; *Q*_array_, and *Q*_M_ represent the respective constant phase element of cavity array and membrane as a component for simulating capacitive properties of the electrode.

Microcavity arrays (Scheme 1) were prepared on a 1 cm^2^ geometric area electrode comprised of one-sided gold deposited on silicon wafer substrate (100 nm gold layer on silicon wafer), (Maher et al., 2016; Basit et al., 2017; Gimenez et al., 2020; Robinson et al., 2020; Sarangi et al., 2022), using PS microsphere templating. The SEM image of the gold electrode confirms formation of continuous ordered pore array (Supplementary Figure S2) across the gold substrate.

The array was then functionalized with SAM of 6-mercaptohexanol at the top surface of the array to help stabilize the lipid monolayer, as reported previously (Maher et al., 2016; Basit et al., 2017; Gimenez et al., 2020; Robinson et al., 2020; Sarangi et al., 2022) and described in detail in the earlier section (Maher et al., 2016; Basit et al., 2017; Gimenez et al., 2020; Robinson et al., 2020; Sarangi et al., 2022). Thereafter, the pores are buffer filled by sonication and a lipid monolayer assembled by Langmuir Blodgett deposition to form the proximal leaflet followed by vesicle fusion to form the distal monolayer to complete the bilayer. To isolate glycosphingolipids to the outer leaflet of the membrane, in analogy to the mammalian plasma membrane, we incorporated the GSLs only into the vesicle used for liposome fusion.

### Electrochemical impedance spectroscopy (EIS)

**FIGURE 1:**
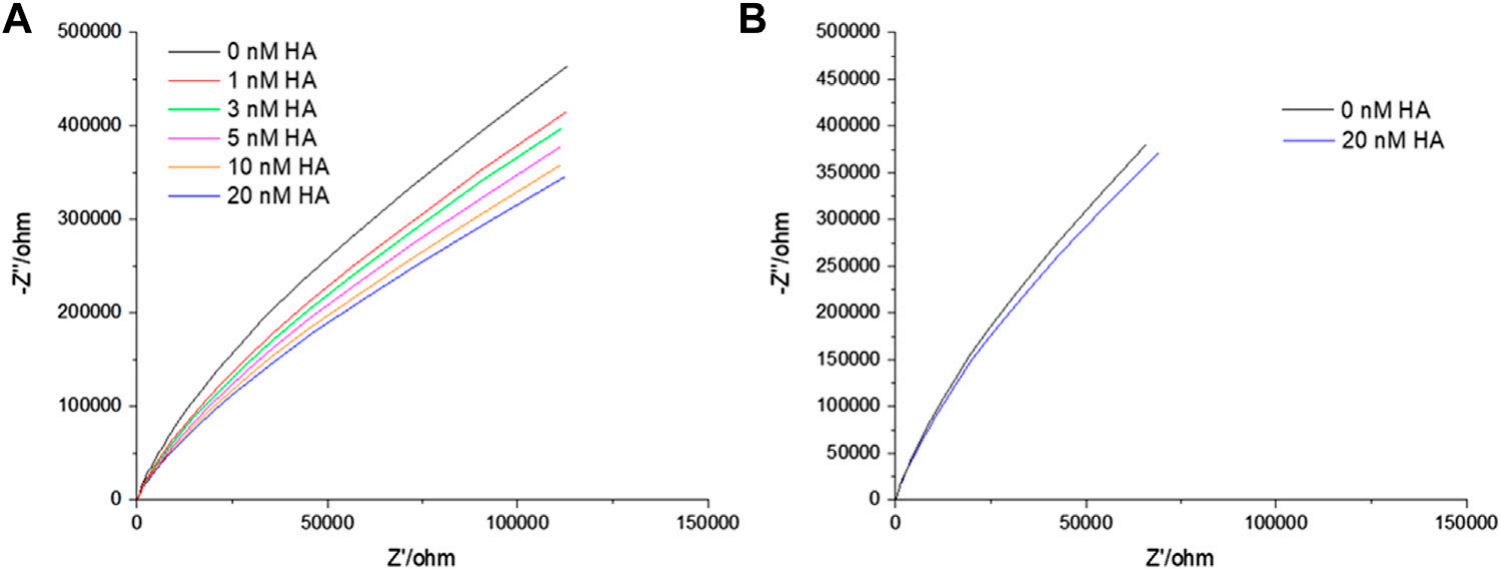
Representative Nyquist plot titration of HA into contacting solution at a DOPC bilayer doped with 1 mol% GD1a suspended across 1 μm cavity arrays in PBS buffer at pH 7.4. An incubation period of 30 min was established for each of the concentrations. All EIS measurements are performed in 0.01 M PBS within the frequency ranges between 0.05 Hz to 10^5^ Hz at 0 V DC bias potential versus Ag/AgCl (1M KCl) with an AC amplitude of 0.01 V. In the EIS setup, gold supported MSLB served as working, Ag/AgCl (1 M KCl) as reference and Pt wire as counter electrode.

EIS is a label-free technique and very sensitive to variations in membranal environment. One can obtain relative quantitative insight by extracting the bilayer resistance values (*R*_M_) through fitting the EIS data to the equivalent circuit model (ECM) which is presented in Scheme 1. Supplementary Figure S3 shows the formation of stable bilayer at the gold electrode. The structure of all three GSLs (GD1a, GM3 and GM1) are shown in Supplementary Figure SI, S4. Figure 1A represents the non-Faradaic impedance Nyquist plots (Z″ vs. Zʹ) obtained from DOPC bilayer containing 1 mol% of GD1a at different concentrations of HA. Figure 1B shows the control experiment without GD1a in the bilayer. Although, the convention in EIS for Nyquist plots is to have matching scales on x and y axes, so we have shown the data according to convention in SI materials (Supplementary Figures S5A,B).

**FIGURE 2:**
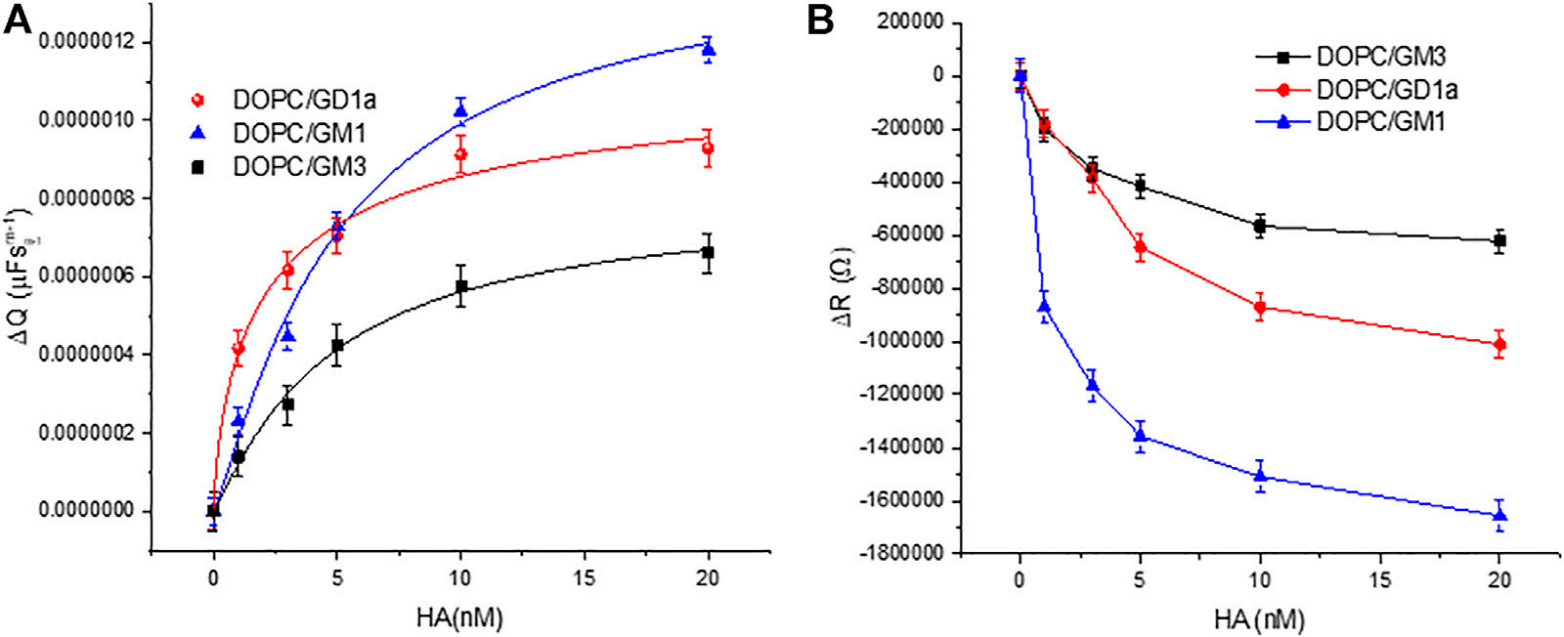
Relative variation of the DOPC bilayer **(A)** capacitance (ΔQ) and **(B)** resistivity (ΔR) on varying HA concentration in bilayer contacting solution. The external leaflet of the bilayer contains 1 mol% of the indicated glycolipids. All EIS measurements were performed within frequency range 0.05 Hz–10^5^ Hz at a DC bias voltage of 0 V with an AC amplitude 0.01 V in 0.01 M PBS at 22 ± 1 °C. The data are shown as means ± SD (*n* = 3).

The change to the DOPC bilayer capacitance (ΔQ) and resistivity (ΔR) on incubation of HA at varying concentration with bilayers containing 1 mol% of different glycolipids are shown in Figures 2A,B respectively. As the experimental window for the protein binding studies is 3.5–4 h, we confirmed in control experiments that the bilayers are stable beyond this experimental window (Supplementary Figure S3). The structure of all three GSLs (GD1a, GM3 and GM1) are shown in Supplementary Figure S4. We also performed control measurements to understand if there was any evidence of non specific association of HA with the membrane in absence of gangliosides (Supplementary Figure S5).

**FIGURE 3:**
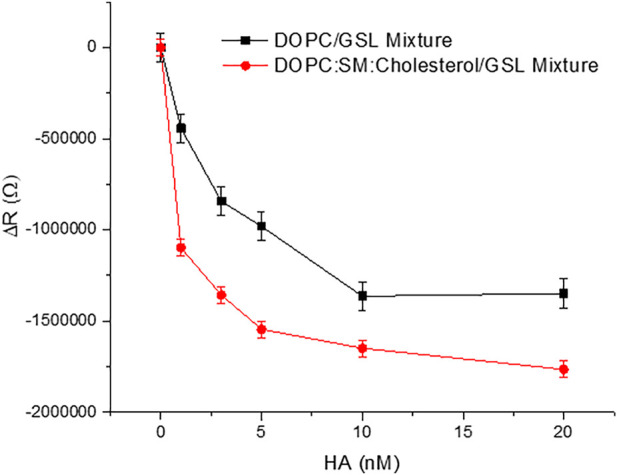
Representative resistance changes on HA binding to GSL mixture (1 mol%) at MSLBs of simple DOPC and in domain forming lipid compositions [DOPC:SM:Cholesterol (4:4:2)]. The data are shown as means ± SD (*n* = 3).

Figure 3 shows representative plots of the membrane resistance changes on incubation of HA with the GSL mixture (1 mol%) at DOPC MSLBs and in domain forming lipid compositions [DOPC:SM:Cholesterol (4:4:2)]. Supplementary Figures S6A,B) shows the variation in relative capacitance and resistance upon HA incubation with the MSLB composed of DOPC composition with 1 mol% and 5 mol% GSL mixture respectively.

### Fluorescence lifetime correlation spectroscopy (FLCS) and fluorescence lifetime imaging microscopy (FLIM)

**FIGURE 4:**
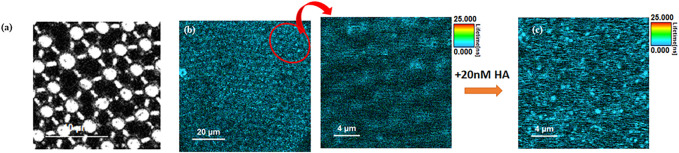
Representative **(A)** reflectance and **(B)** Left FLIM images obtained from MSLB containing a DOPC:SM:Chol + GSL mixture. The upper leaflet of the membrane was stained with 0.01 mol% of ATTO-655 DOPE. Right, A zoomed in area (indicated in oval in **(B)**) is shown prior to HA addition. **(C)** shows a FLIM image obtained from the fluorescently labeled HA protein (20 nM HA-ATTO532) obtained from the identical region to **(B)**.

FCS was employed to assess the lateral diffusivity of fluorescently labelled full-length viral protein HA (HA-ATTO532) on a PDMS-MSLB platform, and its interaction with individual GSLs and mixed GSL was examined at both DOPC and DOPC:SM:Chol membrane compositions. Representative fluorescence lifetime images (FLIM) without and with 20 nM HA at DOPC:SM:Chol+1%GSL MSLB are shown in Figure 4.

FLIM imaging (Figure 4) was used to confirm formation of a continuous spanning lipid bilayer. These are consistent with other studies that similarly show that on buffer filled gold arrays of 1 μm or less pore diameter, a continuous bilayer is formed across the array that is stable for roughly 15 h (Mallon et al., 2010; Jose et al., 2011; Berselli et al., 2021). FLIM was used to confirm bilayer formation across aqueous-filled pores at the PDMS substrates. With much larger pore diameters in PDMS (∼2 μm) and the relative hydrophobicity of this surface we do occasionally see pores that failed to aqueous fill, and are without bilayer but the FCS measurements unlike gold, are pore by pore so imaging/FCS is only collected at bilayer spanning pores unfilled/spanned pores are easily distinguished in microscopy. Figure 6A represents ACFs obtained for labelled DOPE-ATTO655 incorporated into DOPC (black circles) and DOPC:SM:Chol (4:4:2) (blue circles) MSLBs doped with mixed GSL (1 mol%). Figure 6B ACFs for labelled DOPE-ATTO655 in presence (red circle) and absence (blue circle) of 20 nM HA in ternary composition of [DOPC:SM:Chol (4:4:2)] MSLB.

### Inhibition study monitored by label free EIS technique

**SCHEME 2:**
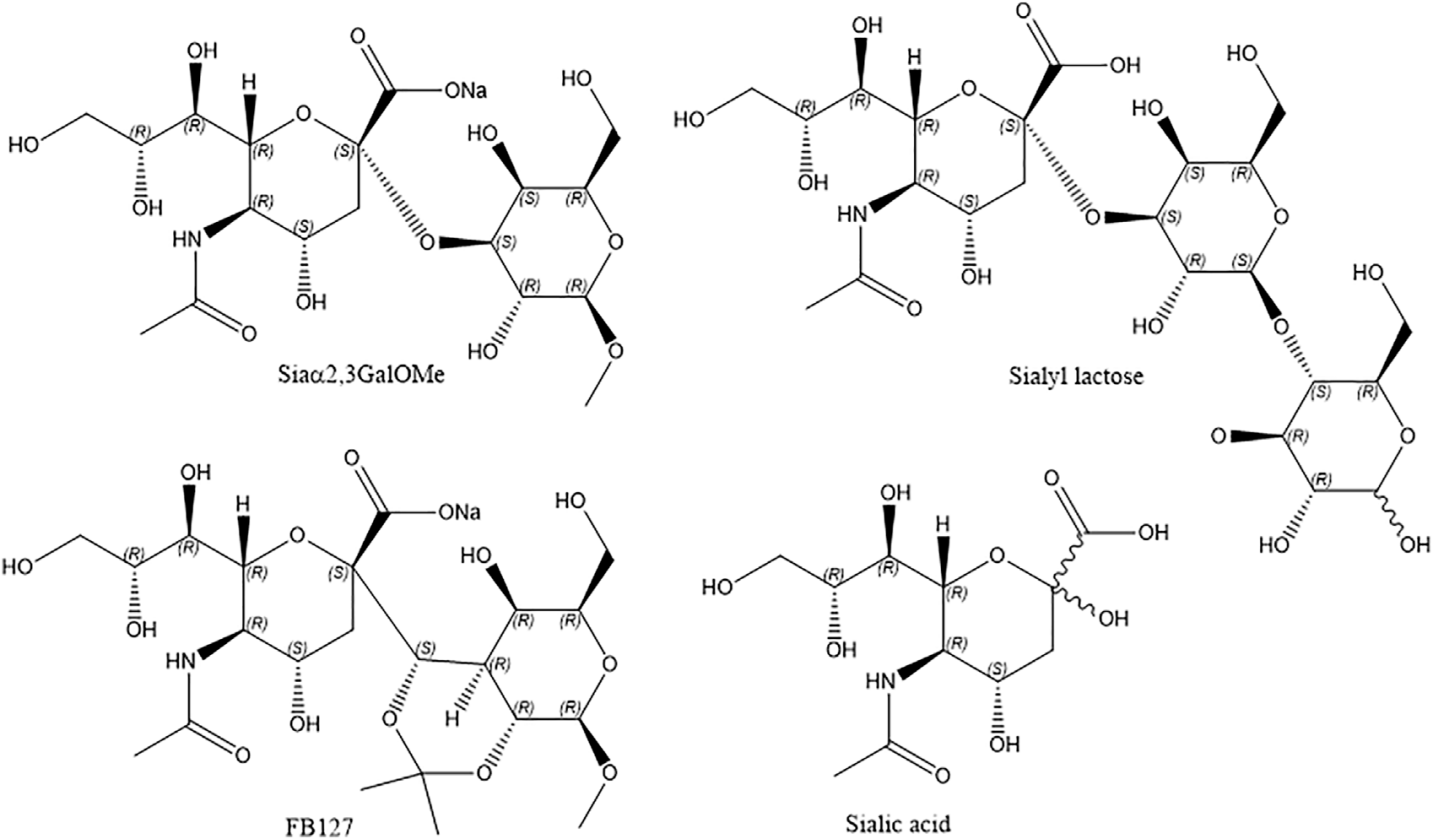
The chemical structure of inhibitors reported here.

Having established GSL-mediated HA binding at the membrane, we then investigated the inhibitory influence of synthetic sialic acid-containing inhibitors (Scheme 2) on HA-GSL binding and compared the response with sialic acid and human milk oligosaccharide sialyl lactose as a HA entry inhibitor.

Figure 7 and Supplementary Figure S8 represents the plots of variation of ΔR and ΔQ of the ternary composition bilayer with and without the drugs or inhibitors. The details of the synthesis of Siaα2,3-GalOMe and FB127 and their NMR, are given in the Supporting Information (Supplementary Schemes S1,S2).

## Discussion

### Impact of GSL and multivalent influenza full viral HA protein recognition on electrochemical properties of the MSLB

EIS is a label-free technique and very sensitive to variations in membranal environment. We used this method to investigate the variation in bilayer resistance and capacitance upon treatment of the membrane with the full viral protein HA. At first, we evaluated the affinity of HA towards the most fluidic model membrane; DOPC lipid bilayers doped with 1 mol% of different individual GSL receptors; GM1, GM3 and GD1a at gold array supported MSLBs. Figure 1 represents the non-Faradaic impedance Nyquist plots (-Z″ vs. Zʹ) obtained from DOPC bilayer containing 1 mol% of GD1a at different concentrations of HA. If the bilayer impedance is increased, the data in the Nyquist plot shifts toward -Z″ (*y*-axis) whereas the shift toward Zʹ (*x*-axis) is an indication of a reduction in bilayer impedance or increased admittance (Daniels and Pourmand, 2007; Skalová et al., 2018; Sarangi et al., 2020). In the latter case, this implies enhanced permittivity of the membrane toward ions which can be due to perturbation in lipid packing in the bilayer (Daniels and Pourmand, 2007; Skalová et al., 2018; Sarangi et al., 2020). One can obtain relative quantitative insight by extracting the bilayer resistance values (*R*_M_) through fitting the EIS data to the equivalent circuit model (ECM) which is presented in Scheme 1. The ECM circuit consists of *R*_el_ (electrolyte resistance) and *C*_stray_ (stray capacitance) which are in parallel, *C*_stray_ accounts for the electrochemical cell, cable or connector. Further, the *R*_*el*_ and *C*_stray_ remain in series with a parallel combination of resistor (*R*_M_) and a constant phase element (CPE) (*Q*_M_) that reflects the electric and dielectric properties of bilayer formed on the electrode surface respectively. *R*_M_ and *Q*_M_ are in series with each other and in parallel with cavity array resistance (*R*_array_) and CPE of microcavity array (*Q*_array_) (Sarangi et al., 2020). In the equivalent circuit, we have chosen CPE over pure capacitors because of the microscopic heterogeneity of the working electrodes arising from the porous microcavity arrays and the assembled lipid bilayer is itself inhomogeneous in nature. The relative variation in membrane resistance and capacitance values before and after the incorporation of HA can be extracted from the fitting of the EIS data. As described previously, (Berselli et al., 2020; Sarangi et al., 2020), we report normalized resistance and capacitance values for different systems. In this way, we can compensate for any deviation in absolute resistance and capacitance values due to small variations in the uniformity of cavity packing and electrode dimensions which result from the substrate fabrication method (Sarangi et al., 2020). As the experimental window for protein binding studies at MSLB is 3.5–4 h, we have confirmed that prior to protein titration, the bilayer remains stable throughout this time-period (Supplementary Figure S3).

Typical absolute resistance and capacitance of DOPC over MSLB were measured as 2.64 ± 0.2 MΩ and 5.03 µFs^m−1^±0.3 respectively from the EIS studies. The values lie within reported ranges for related biophysical models (Khan et al., 2013, 2016; Sarangi et al., 2022). Here, the observed resistance and capacitance values have not been normalized to the electroactive surface area because of uncertainty in the membrane and electrode area arising from variation in the electrode area or roughness from substrate to substrate. Such variation is expected during substrate fabrication due to variation in packing in PS sphere over 1 cm × 1.1 cm flat gold electrodes which results in around a 2–5% variation in the electrode area/roughness as described recently (Sarangi et al., 2022).

Figure 1 represents the Nyquist plots of DOPC + GD1a bilayers before and after binding to different concentrations of HA (0–20 nM). With increase in HA concentration, the impedance is decreased reflected in the Nyquist trace shift towards the *x*-axis. The variation in bilayer resistance (ΔR) after HA incorporation was determined relative to the initial bilayer resistance at 0 nM HA. Notably, incubation of trimeric HA with the GSL-containing membrane induced a marked and systematic reduction in membrane resistance and increase in capacitance (Figure 2) with increasing HA concentration. Enhanced membrane capacitance indicates decreasing bilayer thickness or may also reflect changes to bilayer roughness (or curvature) upon HA binding. This response notably contrasts with our earlier observation for the homomeric HA1 subunit association with the membrane (Berselli et al., 2020). The increase in charge transfer resistance during HA1 subunit binding was attributed to reduced electron transfer to the electrode surface through the resulting interfacial membrane bound HA1 layer. The extended full length protein’s dielectric properties may account for this contrast and the insertion of HA subunit into the membrane may also play a role, as unlike HA1 it contains the hydrophobic stem domains which may interact with membrane altering lipid packing and increasing admittance pointing to potential cooperative interaction with the bilayer on GSL association (Jahn and Sudhof, 2000; McMahon and Gallop, 2005; Gallop et al., 2006; Zimmerberg and Kozlov, 2006; Spasov et al., 2008; Ge and Freed, 2009).

To estimate an empirical apparent equilibrium dissociation constant K_D,_ for the association of HA with the individual embedded receptors in DOPC MSLBs, HA was titrated into the membrane contact solution over the concentration range 1–20 nM and membrane impedance was monitored. We used the Hill-Waud binding model (please see SI) to fit the ΔQ values for the interaction of protein and receptor (Shi et al., 2007). GD1a has the lowest K_D_ value (2.86 ± 0.04nM, n = 1.39 ± 0.6) followed by GM3 (4.68 ± 0.06nM, *n* = 1.01 ± 0.1) and GM1 (5.017 ± 0.05nM, *n* = 1.17 ± 0.3), indicating that the GD1a and GM1 have the highest and lowest affinity for HA, respectively. The difference in affinity can be attributed to the difference in number and position of oligosaccharides in each GSLs. In our previous report on the HA1 subunit, we estimated the K_D_ value for GD1a as 17.47 ± 2nM, *n* = 2.56, for GM3 as 23.96 ± 4nM, *n* = 1.41 and for GM1; 41.3 ± 9nM, *n* = 1.81 (Berselli et al., 2020). Indicating as expected, that the full trimeric protein HA has higher affinity toward the host membrane compared to its single subunit. Here, the structure of all three GSLs (GD1a, GM3 and GM1) are composed of the same ganglio-tetrose backbone with 1 or 2 sialic acid (SA) residues (SI, Supplementary Figure S4). Both GM3 and GM1 possess one SA residue, but it is an internal residue in GM1, whereas for GM3 it is a terminal residue due to the absence of final two carbohydrates of the tetrose backbone. GD1a also possesses a similar internal SA residue as GM1, but it also has an extra terminal SA, which may promote affinity for HA protein compared to GM1 and GM3. The data overall indicates that the head group structure of each GSL in the membrane can influence their association with the viral glycoprotein HA. Related GSL structural dependence on the binding of the Sendai viral protein was recently reported at supported lipid membrane models (Lam et al., 2022).

Besides, although there is a modest difference between the Hill coefficients (n-value) determined herein for the full-length HA compared to the monomeric unit HA1. Both, however, are greater than 1 and both indicative of positive cooperative binding. It is notable that the observed range of Hill coefficients for the full trimeric HA protein is comparable to the reported values of full-length HA protein measured by other methods (Srinivasan et al., 2008, 2013).

In our investigation, the difference in the magnitude of membrane resistance change (ΔR) for each GSL upon HA association is quite significant and may be due to the influence of ganglioside packing in influenza viral protein HA association but also likely indicates direct interaction of HA with the membrane. We also performed control measurements to understand if there was any evidence of non-specific association of HA with the membrane in absence of gangliosides (Supplementary Figure S5).

As shown in (Supplementary Figure S5), incubating HA with DOPC bilayer without GSL showed no impedance change within the experimental error. Our study, therefore, indicates that HA binds to the model membrane specifically through GSL association. Given that the binding of viruses to the host membranes relies on many weak specific interactions, the absence of nonspecific interactions is particularly crucial in this context. In a previously reported simulation, it was predicted that GM1 and GM3 will pack differently in the bilayer dictated by differences in their associated headgroups (Gu et al., 2017). Moreover, it was reported that as the HA proteins do not significantly alter their conformation after binding (Sauter et al., 1992) under neutral pH conditions, the differences in interaction or adhesion affinity may be attributed to multivalent binding of the influenza HA viral glycoprotein at the SLB’s SA receptors (Lee et al., 2016). We have further established the influence of HA multivalency through FCS studies, which will be discussed in the FCS section.

Next, we evaluated the role of membrane composition and receptor density on HA binding. We initially compared the relative variation in ΔR after titrating different concentrations of HA with the MSLB containing 1 mol% of GSL mixture (total extract containing GM1, GD1a, GD1b, GT1b) doped at the homogeneous DOPC bilayer with that of a ternary, domain forming composition composed of DOPC:SM: Chol. The absolute resistance and capacitance of ternary composition (DOPC:SM: Chol) forming MSLB before HA incubation were found to be 4.78MΩ ± 0.9 and 4µFs^m−1^±0.4 respectively (Khan et al., 2013, 2016; Sarangi et al., 2022). DOPC:SM:Chol membranes were found to have a greater absolute resistance value and lower capacitance value compared to DOPC membranes, indicating that they were more densely packed.

Figure 3 plots the relative resistance change to the GSL containing DOPC and DOPC:SM:Chol membrane on HA binding. The magnitude of the changes is ∼24% greater for the ternary composition compared to DOPC. This is tentatively attributed to more favorable binding of HA into domain forming ternary composition compared to DOPC alone. This is in agreement with the reported role of membrane rafts or domain forming compositions in promoting viral attachment (Verma et al., 2018; Ripa et al., 2021). Next, we compared the ΔR changes upon HA incubation with the DOPC MSLB containing two different GSL mixture concentrations, 1 and 5 mol% (). The relative decrease in resistance, ΔR for MSLB with 1 mol% GSL (ΔR_sat_ = 1.9MΩ ± 0.1) is consistently smaller than the change observed at 5 mol% GSL (ΔR_sat_ = 2.4MΩ ± 0.2) (Supplementary Figure S6B). Moreover, it is also observed that the ternary composition containing higher receptor density yields a lower dissociation constant (K_D_∼3.1 nM ± 0.3) than that of lower receptor density (K_D_∼10.5 nM ± 0.5) (Supplementary Figure S6A). This implies, as expected, stronger affinity of HA for the MSLB with the higher % GSL.

### Lateral mobility of the HA−GSL complex and the bilayer- investigating the influence of different GSLs on HA binding with host membrane, HA multiplicity

Fluorescence lifetime correlation spectroscopy (FLCS) was employed to assess the lateral diffusivity of fluorescently labelled full-length viral protein HA (HA-ATTO532) on a PDMS-MSLB platform, and the interaction with individual GSLs and mixed GSL was examined at both DOPC and DOPC:SM:Chol membrane compositions. Representative fluorescence lifetime images (FLIM) without and with 20 nM HA at DOPC:SM:Chol+1%GSL MSLB are shown in Figure 4. As seen in the reflectance image (Figure 4A), the cavities are buffer filled and FLIM images (Figure 4B), shows continuous MSLB formation over the buffer filled PDMS array. FLIM was acquired by z-scanning to focus on the optimal MSLB plane across the buffer filled cavity array, yielding maximum fluorescence intensity counts. The point FLCS measurements are performed at these foci at the center of the micropore cavity array to obtain the diffusion of both lipid and protein.

**FIGURE 5:**
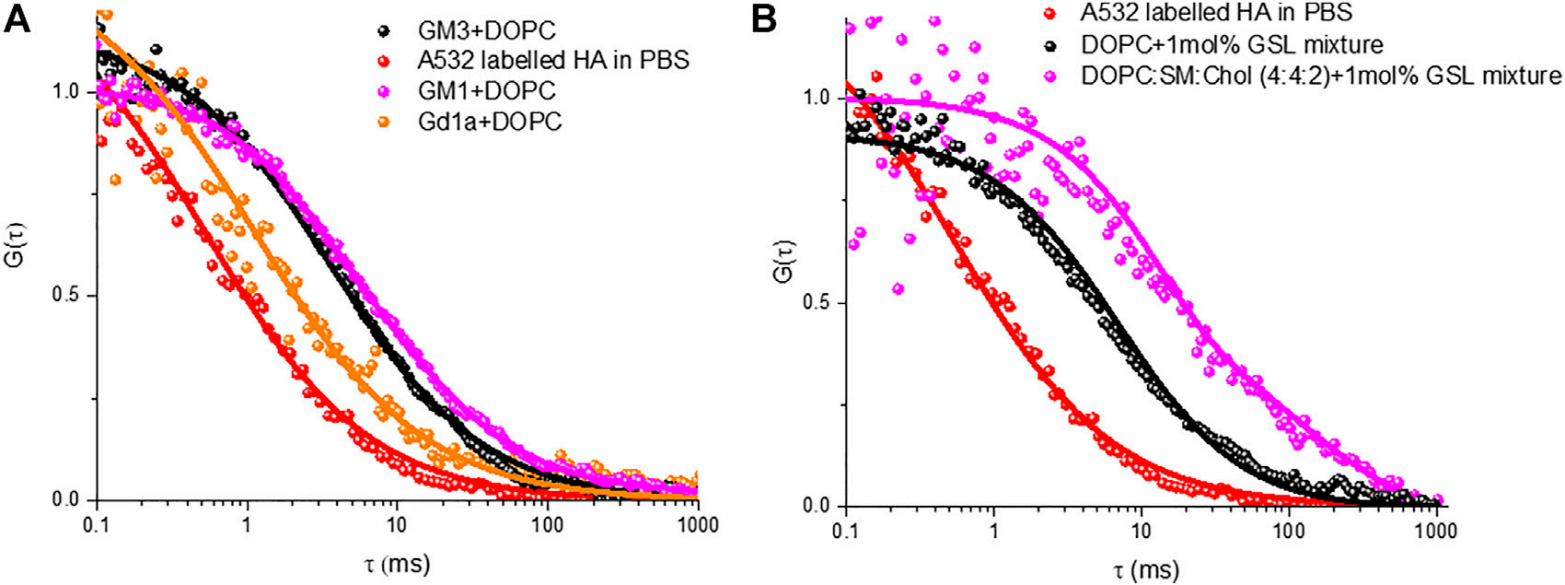
Representative FLCS raw (symbol) and fitted (solid line) autocorrelation functions (ACFs) obtained for labelled HA-ATTO532 after binding with different MSLBs comprised of **(A)** DOPC containing 1 mol% of different individual GSLs namely GM3 (black) GM3 (magenta) and GD1a (orange), **(B)** 1 mol% of mixed GSL containing DOPC (black) and DOPC:SM:Chol (4:4:1) (magenta) MSLBs. In each panel, the free HA-ATTO532 diffusing in PBS solution are shown in red.

Figure 5A shows representative ACFs for HA-ATTO532 after incubation with different MSLBs composed of DOPC + GD1a (1 mol%) (black circles), DOPC + GM1 (1 mol%) (green circles), and DOPC + GM3 (1 mol%) (blue circles). The ACFs of HA-ATTO532 at different GSL-doped MSLB were fit to the 2D diffusion model (Eq. 2), and the lateral diffusion coefficients were calculated for DOPC + GD1a, DOPC + GM1 and DOPC + GM3 as ∼7.7 ± 0.3 μm^2^ s^−1^ for, ∼3.92 ± 0.5 μm^2^ s^−1^ and ∼4.1 ± 0.3 μm^2^ s^−1^ respectively. We obtained an anomalous exponent (α) ∼1 across all compositions indicating Brownian diffusion. The differences in the lateral diffusion coefficients of HA associated with each of the three different glycolipids is significant. It is also noteworthy to compare the values to the diffusion coefficients of the HA1 sub unit bound to the same glycolipid, which were previously measured to be 13 µm for DOPC + GD1a and 5 μm^2^ s^−1^ for DOPC + GM1 and DOPC + GM3. Notably, the trend in the data is consistent, with the fastest diffusion observed for the GD1a glycolipid, but all three diffusion coefficients are slower than recorded for HA1.

Therefore, from the diffusion data, it is evident from our present investigation and previous study that the binding of trimeric HA protein with host cell membrane differs from that of monomeric HA1 subunit, which we attribute to differences in the hydrodynamic radius of the membrane associated HA-GSL complexes. Furthermore, the lateral diffusion coefficient suggests that the hydrodynamic radius of the membrane associated HA-GSL complex for GD1a is not the same as for the other two GSLs. Therefore, we have estimated the radius of the final HA-GSL complex by using the Saffman-Delbrük (SD) model (SI) which yields a value of 0.6 nm for the HA-GD1a complex, which correlates well with a diffusion of two GD1a lipids. For the membrane associated complex formed by GM1 and GM3 on the other hand we estimate radii of 8.9 and 7.9 nm, respectively. Because HA is a homotrimer protein with a maximum of three binding sites, the differences in size/diffusion value might be ascribed to multivalency. The exact relationship between IAV valency and diffusion coefficient is not fully known, but existing theoretical studies indicate that higher diffusion coefficient will correspond to lower mean valency and vice versa (Block, 2018). Our previous study with a monovalent HA1 subunit estimated a radius of 0.3 nm for GD1a and 7.0 nm for its assembly with GM1/GM3. Therefore, our present study with the full influenza HA may suggest higher valency of the full protein with GSL however, as described the hydrophobic stem of this protein may directly interact with membrane on binding, which could also contribute to the observed difference in membrane bound radius of the GSL-HA complex.

**FIGURE 6:**
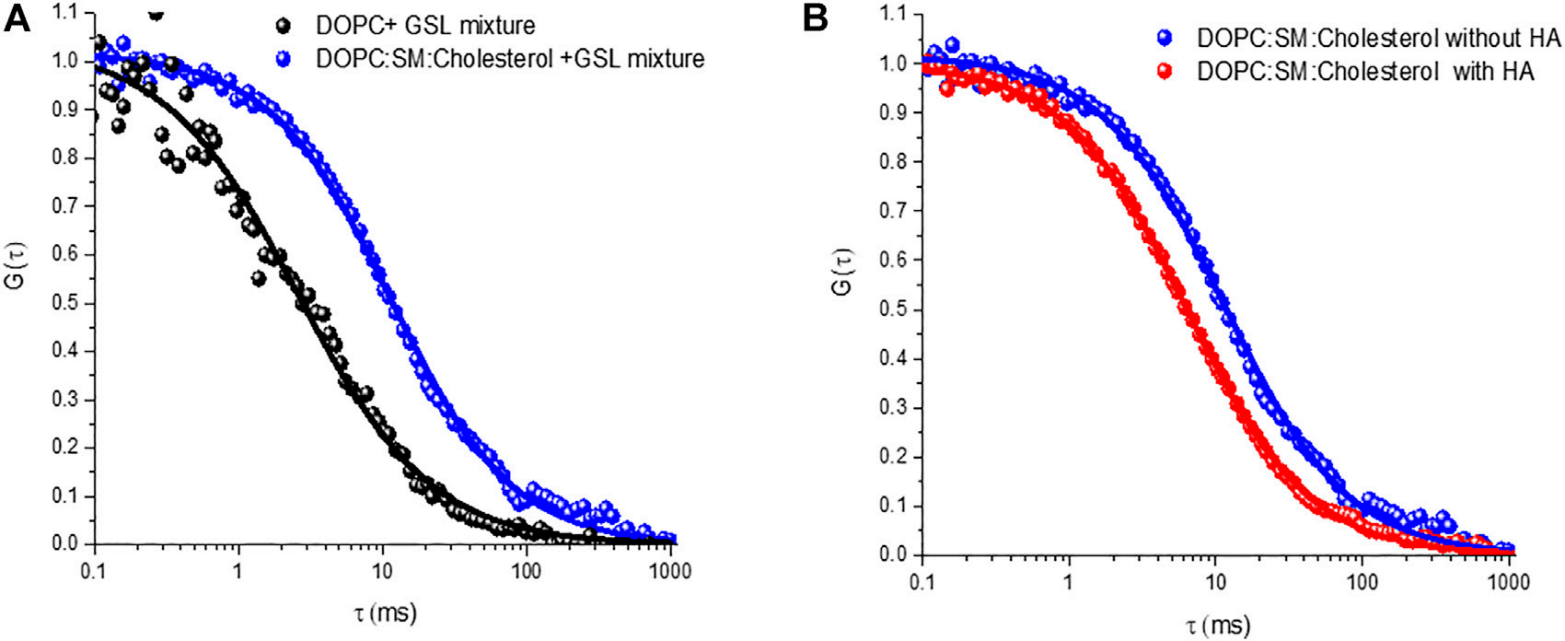
Representative autocorrelation functions (ACFs) obtained for labelled DOPE-ATTO655 **(A)** incorporated into DOPC (black circles) and DOPC:SM:Chol (4:4:2) (blue circles) MSLBs doped with mixed GSL (1 mol%). **(B)** ACFs for labelled DOPE-ATTO655 in presence (red circle) and absence (blue circle) of 20 nM HA in ternary composition of [DOPC:SM:Chol (4:4:2)] MSLB.

Nonetheless the SD calculated radius agrees well with the radius expected for diffusion of two GD1a lipids. Trimeric HA has in principle, the ability to bind up to three GSLs and this may account for the slower diffusion of the HA-GM3/GM1 complexes compared to that of the HA-GD1a complex and compared to monovalent subunit, HA_1_ (Berselli et al., 2020). As there is significant evidence that ordered membrane domains promote HA-glycan binding, (Hess et al., 2005, 2007; Polozov et al., 2008; Engel et al., 2010), we examined the influence of the ternary membrane composition on HA association to 1 mol% GSL mixture (pink ACF in Figure 5B). The diffusion coefficient of lipid marker DOPE-ATTO655 in the ternary composition in the absence of viral glycoprotein HA (Figure 6A) was determined as 3.7 ± 0.6 μm^2^ s^−1^ which as expected, is significantly slower than the value obtained for DOPC only (10 ± 0 7 μm^2^ s^−1^). We then compared HA diffusivity on incubating the labelled viral protein (HA-ATTO532) at DOPC, and DOPC/SM/Chol (4:4:2) (mol/mol/mol) doped with 1 mol% GSL mixture at the distal leaflet (Table 1). The lateral diffusion coefficient for labelled HA was obtained as ∼1.5 ± 0.6 µm for ternary domain forming composition which is dramatically slower than the diffusion coefficient of HA bound to DOPC/GSL of ∼4.8 ± 0.2 μm^2^ s^−1^.

**TABLE 1:** Lateral diffusion coefficients of labelled HA-ATTO532 obtained after binding to lipid membranes containing mixed GSL in DOPC and ternary MSLB systems, from FLCS. The lateral diffusion of DOPE-ATTO655 decreases with membranes fluidity, here expressed by the DOPE diffusivity.

System	*D* (μm^2^ s^−1^) HA-ATTO532	*D* (μm^2^ s^−1^) DOPE-ATTO655
DOPC:SM:Chol +1 mol% GSL	1.5 ± 0.6	3.7 ± 0.6 (no HA), 7.7 ± 0.5 (20 nM HA)
DOPC+1 mol% GSL	4.8 ± 0.2	10 ± 0.7 (no HA), 10 ± 0.6 (20 nM HA)

The anomalous exponent (α) is ∼1 for all the compositions.

The diffusion coefficient of HA-ATTO532 in a DOPC bilayer is approximately half that of the DOPE lipid marker (∼10 μm^2^ s^−1^) and even lower in the domain forming composition (∼3.7 ± 0.6 µm). A similar effect was observed for the HA1 subunit, implying that influenza viral glycoprotein HA associates with GSL in the ordered domains of the ternary composition, and this observation correlates with previous theoretical study (Nicolau et al., 2006). Furthermore, the favorable incorporation of HA glycoprotein into the domain forming composition seems to perturb the bilayer membrane organization, (McMahon and Gallop, 2005; Gallop et al., 2006), resulting in fluidization, as evident from both the FCS and EIS studies. It is reported that the lipid-packing perturbation is caused by the insertion of protein into the outer leaflet, that behaves like a wedge to perturb the overall lipid packing (McMahon and Gallop, 2005; Gallop et al., 2006; Zimmerberg and Kozlov, 2006; Spasov et al., 2008; Ge and Freed, 2009). The wedge-shaped lipid DOPE-Atto655 preferably partitions into the liquid-disordered phase, (Arita et al., 2015; Talbot et al., 2019), and this results in the observed increase in diffusion coefficient of the lipid (DOPE) when HA is incorporated into the domain forming MSLB (Figure 6B).

### Anti-influenza entry inhibitors or drugs screening study in MSLB

Having established GSL mediated HA binding at the membrane, we then investigated the inhibitory influence of synthetic sialic acid-containing inhibitors (Scheme 2) on HA-GSL binding, and compared the response with sialic acid and human milk oligosaccharide sialyl lactose as a HA entry inhibitor. Addition of substances that inhibit virus binding are expected to cause virus attachment to cells and resulting uptake to decrease, by binding to virus surface receptors and reducing their affinity for the membrane SAs.

**FIGURE 7:**
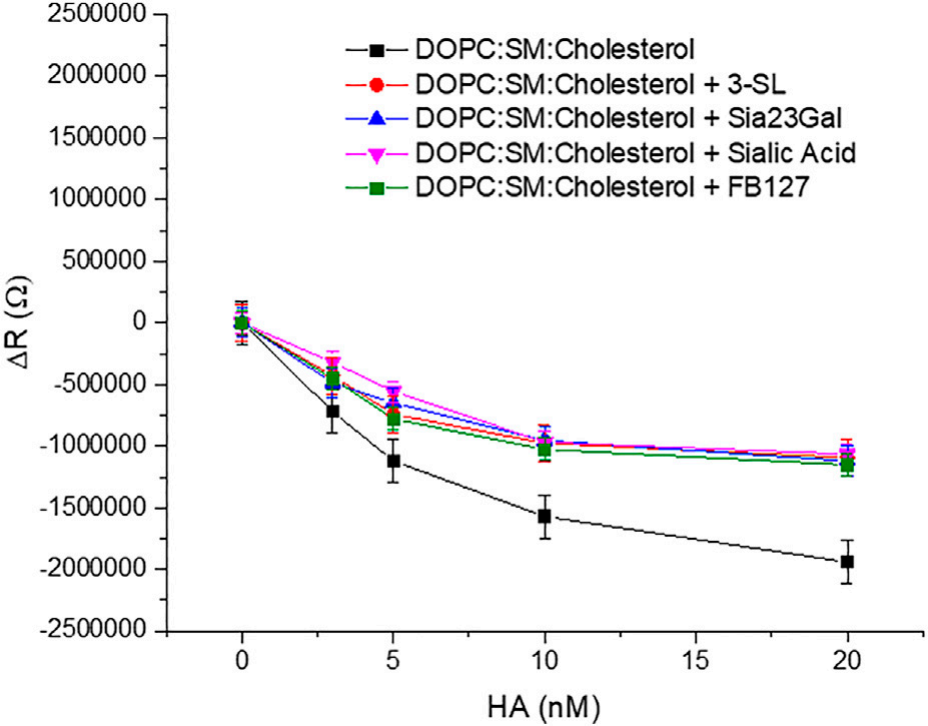
Representative plots of showing variation of ΔR of the ternary composition bilayer with and without the drugs or inhibitors (∼16 mM).

EIS experiments were performed on the domain forming membrane composition in the presence and absence of the respective inhibitors to understand if a background of inhibitor reduced membrane binding affinity of HA. Approximately 16 mM of inhibitor was used which is well above the previously reported inhibitory concentration of other sialic acid derivatives (Sauter et al., 1989). We selected a high background inhibitor concentration to maximize any inhibition by the drugs as we anticipated the response may be quite weak. The inhibitors were preincubated with HA for 30 min to ensure maximal drug-HA binding. Then, these inhibitors bound to HA were introduced into the MSLB contacting solution. For convenience, we kept the concentration fixed for each inhibitor and compared the relative variation of ΔR of the domain forming bilayer with and without the inhibitors (Figure 7). We ensured that no osmotic imbalance occurred in the electrochemical cell and maintained inhibitor concentration fixed (16 mM) at the electrolytic solution throughout the EIS experiment.

The data show clear evidence that the synthetic compounds Siaα2,3GalOMe, FB127, along with human milk oligosaccharide 3′-SL exerts an inhibitory effect towards HA viral protein in the model system. The level of inhibition is similar to that of sialic acid at the concentrations determined. Affinity constants for the inhibitors tested here are in the mM range and consistent with previous literature reports, when they were evaluated by different methods (Ji et al., 2017). FB127 is a recently patented (Woods et al., 2017) inhibitor of influenza HA and a mimetic of the 3′SL and the C-disaccharide derivative Sia23Gal. We selected the 2,3-linked disaccharide as it has similar affinity at the level of a monomeric interaction with the 2,6-linked sialyl lactose and disaccharide according to previous reports (Sauter et al., 1989; Ji et al., 2017).

Evidence of blocking of the HA-GSL interaction is reflected in the reduced resistivity (ΔR) (Figure 7) and ΔQ (Supplementary Figure S8) of the bilayer membrane in presence of an inhibitor. The relative change in ΔR value (ΔR = 1.9 MΩ) with 20 nM HA incubated at the ternary composition (DOPC:SM:Cholesterol) MSLB is lower in presence of different inhibitor (ΔR = 1.06–1.11MΩ ± 0.07). Correspondingly, K_D_ is modestly higher in the presence of inhibitor at ∼1.3 to 3 ± 0.02 nM compared with ∼1 ± 0.07 nM) in their absence. Hence, taken together the inhibitors do show modest inhibition of HA -GSL binding when present at millimolar background concentrations.

This EIS study for the inhibitor screening were carried out to check if our platform is capable of responding to inhibition or not. As discussed, we did see partial inhibition for the HA binding with the host membrane in presence of those inhibitors and moreover, the affinity constants for the inhibitors consistent with previous literature report those are evaluated by different methods (Ji et al., 2017). Overall, the data indicate that the MSLB platform can be utilized as a drug or inhibitor screening platform against the human influenza HA viral protein. A more detailed study where inhibitor concentration will be varied to calculate K_D_/IC50 values in terms of the inhibitor interaction will be carried out in future with a view to new HA inhibitor identification. The details of the synthesis of Siaα2,3-GalOMe and FB127 and their NMR, are given in the Supporting Information (Supplementary Schemes S1,S2).

In conclusion, a model of the full influenza HA protein binding at a glycolipid decorated membrane surface was established and FCS measurements combined with label-free EIS used to untangle the influence some of the factors that contribute towards the interaction of the influenza viral glycoprotein HA at host cell membrane. Moreover, potential anti-influenza entry-inhibitors were synthesized and their interaction with HA was also investigated at the platform. The affinity of influenza A, H_3_N_2_, viral glycoprotein HA at a DOPC was observed to vary with identity of glycosphingolipids, GD1a showed highest affinity while GM1 decorated membrane showed the lowest. Interestingly the response of protein binding was opposite to that of the globular headgroup domain alone as reported previously, with decreasing resistance and increasing capacitance observed here. However, as expected, the affinity of the full protein for the membranes was consistently higher than that of HA1. Lower k_D_ value of HA association in the membrane having higher percentage of GSLs was also observed.

The diffusion coefficient of the trimeric HA protein was found to be lower than that reported the monomeric HA subunit HA_1_, which was attributed to the higher valency of the full HA protein. Finally, incubation of HA with oligosaccharide sialyl lactose and other synthetic HA entry inhibitors were observed to modestly reduce the K_D_ of HA for the GSL decorated membrane indicating the inhibitors inhibit but do not prevent HA membrane binding. Overall, our work establishes the advantage of microfluidic supported lipid bilayers (MSLB) as a versatile platform for the detection of full-length influenza HA and investigates the effectiveness of this platform for screening of the prospective inhibitor against influenza HA entry.

## Data Availability

The raw data supporting the conclusion of this article will be made available by the authors, without undue reservation.
