# Supplementary material for: A cell free biomembrane platform for multimodal study of influenza virus hemagglutinin and for evaluation of entry-inhibitors against hemagglutinin
Source: Front Mol Biosci. 2022 Oct 13;9:1017338. doi: 10.3389/fmolb.2022.1017338 (PMC9608630; doi:10.3389/fmolb.2022.1017338)
Supplement: Supplementary file 1 [file DataSheet1.docx]

**Supporting Information**

**A Cell Free Biomembrane Platform for Multimodal Study of Influenza Virus Hemagglutinin and for Evaluation of Entry-Inhibitors Against Hemagglutinin**

Arpita Roy^†^, Sylvester Byrne^‡^, Nirod Kumar Sarangi^†^, Paul V. Murphy^‡^, Tia Keyes^*,†^

^†^School of Chemical Sciences and National Centre for Sensor Research, Dublin City University, Dublin 9, Ireland

^‡^School of Chemistry, NUI Galway, University Road, Galway, Ireland

*Email: [tia.keyes@dcu.ie](mailto:tia.keyes@dcu.ie)

**1. Liposome preparation**

Briefly, in this work, liposome fusion was used to form the distal lipid leaflet of microcavity supported lipid bilayers (MSLBs). To prepare the liposomes, stock solutions of all vesicle components such as DOPC, brain sphingomyelin and cholesterol 50 mg/mL each, and GM1 (1 mg/mL), GM3 (1 mg/mL) and GD1a (1 mg/mL) were prepared in chloroform and stored in sealed glass vials at -20°C. Fluorescence labelled DOPE-ATTO655 was mixed in a ratio of 50000:1 mol/mol with unlabeled lipids for fluorescence correlation spectroscopy (FLCS) studies. For electrochemical measurements, as electrochemical impedance spectroscopy (EIS) is label-free, fluorescent probe was not included during the preparation of liposomes. Aliquots of the appropriate amounts of the stock solutions were mixed in clean amber glass vials and dried under a gentle stream of nitrogen and placed under vacuum for 1h. The lipids were rehydrated in 1 mL of PBS buffer (pH 7.4) and vortexed vigorously for at least 60 s. Large unilamellar vesicles (LUVs) were prepared by extruding the multilamellar vesicle suspension 11x against a polycarbonate membrane (0.1μm pore size) using a mini hand-extruder (Avanti Polar Lipids). The LUVs were diluted to 0.25mg/mL. Liposomes composed of SM were extruded at 45°C, above the SM transition temperature, to guarantee that vesicles are in the fluidic state.


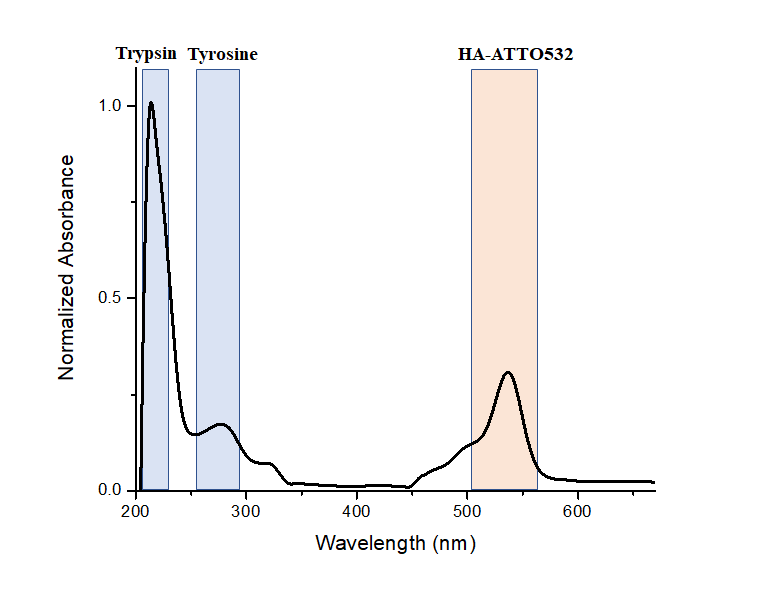


**FigureS1:** Normalized Absorbance spectrum of labelled HA after reaction with ATTO532 NHS-ester. The maximum absorbance of trypsin and tyrosine (blue) with the probe maxima marked in orange has been shown.^1,2^


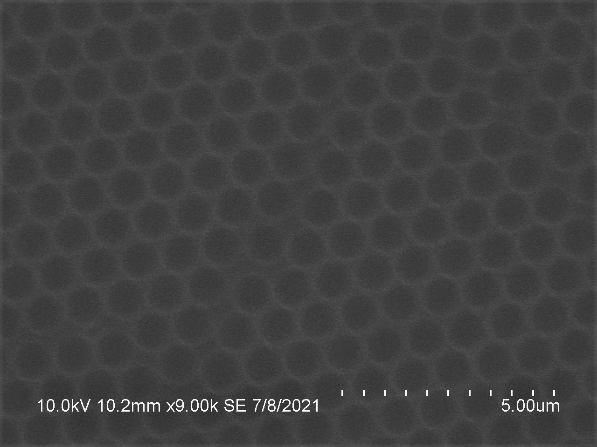

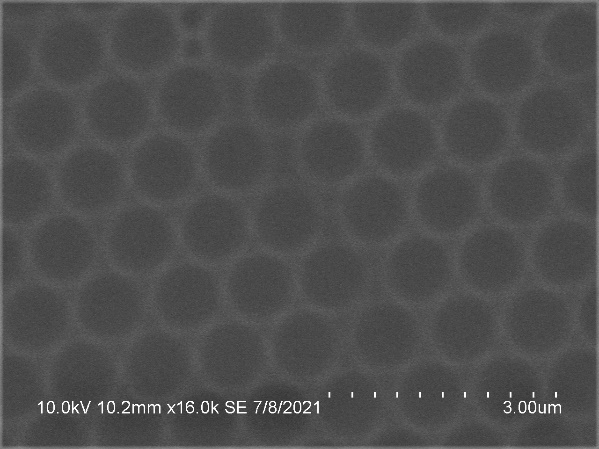


**FigureS2:** SEM image of hemispherical microcavity array obtained from electrochemical deposition through templates assembled from ∼1μm diameter polystyrene spheres on gold substrates.

**2. Hill-Waud binding model:**

The relative change in membrane capacitance from EIS data for different lipid compositions versus HA association concentrations were fit iteratively to the Hill-Waud expression, as defined by equation (S1).

$\Delta Q=\frac{{\Delta Q}_{sat}{(C)}^{n}}{{(K_{D})}^{n}+{(C)}^{n}}$ (S1)

Where, ΔQ is the change in membrane capacitance, defined by $Q_{0}^{M}-Q_{HA}^{M}$, ${\Delta Q}_{sat}$ is change in capacitance at maximum surface loading that relates to the number of available binding sites, $K_{D}$ is the empirical apparent equilibrium dissociation constant, 𝐶 is the concentration of the HA and 𝑛 (dimensionless) is the Hill coefficient of cooperativity.^3^

**3. Electrochemical impedance spectroscopy:**

The electrochemical measurements were performed with a CH760A potentiostat (CH Instruments, USA). A standard 3-electrode cell comprised of gold microcavity suspended bilayer as a working electrode, an Ag/AgCl (1 M KCl) reference electrode and a platinum wire auxiliary electrode. However, herein probe free method is used to avoid interactions with drug in main EIS data. The EIS data were measured over a frequency range of 0.05 to 105 Hz with an ac modulation amplitude of 0.01 V at a potential DC bias of 0 V (vs. Ag/AgCl (1 M KCl)). The DC bias of 0 V was selected for all our EIS measurements in 0.01 M PBS, as there is no electrochemical reaction involved. All measurements were carried out in a glass cell (approximate volume of 4 ml) in contact with PBS buffer maintained at pH 7.4. EIS fitting was done by employing Zview (Scribner Associates, v3.4e) software.

**4.** **Stability of DOPC and DOPC:SM:Cholesterol Bilayer:**

**Figure S3:** Representative resistance obtained for a (a) DOPC and (b) DOPC:SM:Cholesterol bilayer suspended over PBS buffer filled gold cavities. The EIS of bilayer was measured every hour for 5 hours.


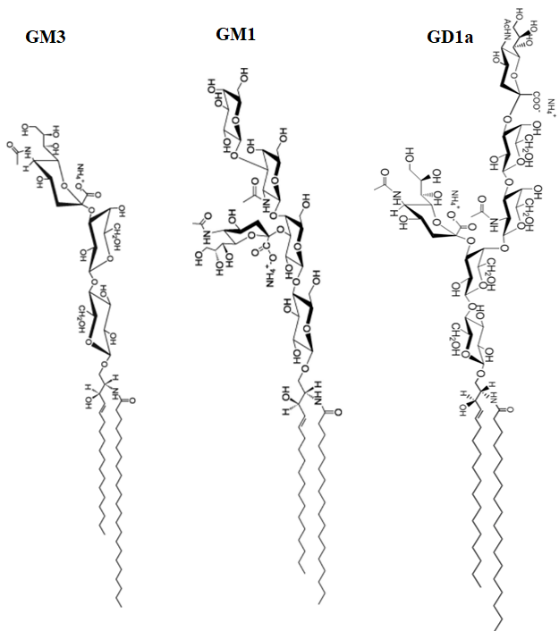


**Figure S4:** The chemical structure of different GSLs that has been studied here.

**5. Binding of HA1 to MSLBs in absence of GSLs:**

Control experiments were carried out to bare lipid membranes comprised raft composition, DOPC/SM/Chol (4:4:2) without GSLs. The results obtained have not shown non-specific binding of HA to MSLBs in absence of GSLs as the membrane resistivity remains constant after exposing the lipid bilayers to HA for 30 min. Figure S3 shows the results obtained for raft lipid bilayer.

**Figure S5:** (a) represents the non-Faradaic impedance Nyquist plots (Z″ vs Zʹ) obtained from DOPC bilayer containing 1mol % of GD1a at different concentrations of HA. (b) shows the control experiment without GD1a in the bilayer.

**Figure S6:** Variation in relative (a) capacitance and (b) resistance upon HA incubation with the MSLB composed of DOPC composition with 1mol% and 5 mol% GSL mixture.

**6. Saffman-Delbrück (SD) model:**

Due to the suspended character of planar MSLBs, where both leaflets are in contact with bulk aqueous phase, we assume the Saffman-Delbrück model (Equation S2) applies and this was used to estimate the diameter of the membrane associated complex.

$D=\frac{KT}{4\pi\mu h}[\ln\left( \frac{\mu h}{\mu^{ʹ}r} \right)-\gamma]$ (S2)

Where is the lateral diffusion obtained 𝐷 from FLCS, 𝐾 is Boltzmann constant, 𝑇 is the absolute temperature, 𝑟 is the radius of the cylindrical membrane inclusion, ℎ is the membrane thickness, 𝛾 is Euler-Mascheroni constant (approx. 0.577), 𝜇 and $\mu^{ʹ}$ are the membrane viscosity and the bulk solution viscosity, respectively. The parameters used to estimate the membrane associated complexes were membrane height ℎ = 3.8 nm, viscosity of the surrounding media $\mu^{ʹ}$= 0.001 Pa.s and membrane viscosity 𝜇 = 0.05 Pa.s.

**FigureS7.** Representative FLCS fitted autocorrelation functions (ACFs) obtained for free ATTO532 (black circles) and labelled HA-ATTO532 (red circles) in PBS (pH 7.4).

**Figure S8.** Representative data showing changes to capacitance (ΔQ) of the of the ternary composition bilayer with and without the inhibitors.

**Scheme S1 Synthesis of Siaα2,3-GalOMe (S5)**

**Scheme S2 Synthesis of FB127 (S12)**

**Compound S4 (SBa236)**

Galactopyranoside **S1**^4^ (0.85 g, 2.28 mmol) and thiosialoside **S2**^5^ (1.60 g, 2.74 mmol) were dissolved in anhydrous CH_2_Cl_2_, concentrated and dried at high-vacuum for 3 h. The mixture was then dissolved in CH_­­3_CN-CH_2_Cl_2_ (10:1) and in the presence of 4Å molecular sieves was stirred at -42 ^o^C for 30 min. NIS (2.36 g, 10.5 mmol) was added to the reaction, and this was followed by dropwise addition of TfOH (0.22 mL, 2.5 mmol). The resulting mixture was stirred at -42 ^o^C for 5h. The reaction was quenched by addition of Et­_3_N (0.418 mL, 0.303 mmol), diluted with CH_2_Cl_2_ (75mL) and filtered over Celite. The filtrate was washed with aqueous 10% Na_2_S_2_O_3_, sodium bicarbonate and was dried over anhydrous Na_2_SO_4_. Flash chromatography (toluene-acetone 6:4) afforded an impure mixture of anomers **S3.** The mixture was taken up into pyridine (15 mL). Acetic anhydride was slowly added to stirred solution to a final concentration of (15 mL) and was continued to stir overnight. Upon completion, the reaction was diluted with EtOAc and washed with 1 M HCl, H_2_O, Brine. The organic layer was dried over Na_2_SO_4_, and the solvent was removed under reduced pressure. Flash chromatography toluene-acetone (7:3) gave the acetylated disaccharides. This mixture was dissolved in EtOAc. A catalytic mixture of 10% Pd-C and 20% Pd(OH)­_2_-C was charged to the solution. The flask was then subjected to 3 purge and refill cycles of hydrogen gas. The heterogeneous mixture was stirred under a positive pressure of H_2_ using a balloon of H_2_ for 16 h. The flask was purged with N_2_, followed by filtration through celite. The filtrate was condensed under reduced pressure. Flash chromatography CH_2_Cl_2_-MeOH (20:1, 9:1) yielded the desired α-linked disaccharide **S4** as a white solid (0.63 g, 39% over 3 steps): ^1^H NMR (500 MHz, cdcl_3_) δ 5.46 (ddd, *J* = 9.3, 5.1, 2.8 Hz, 1H, H-8), 5.33 (dd, *J* = 9.4, 1.5 Hz, 1H, H-7), 5.24 (d, *J* = 8.7 Hz, 1H. NHAc), 4.97 – 4.87 (m, 2H, H-4, H-4’), 4.44 (d, *J* = 7.7 Hz, 1H, H-1’), 4.28 (dd, *J* = 9.8, 3.6 Hz, 1H, H-3’), 4.25 (dd, *J* = 12.5, 2.7 Hz, 1H, H-9a), 4.07 (dd, *J* = 12.6, 5.1 Hz, 1H, H-9b), 4.03 – 3.96 (m, 2H, overlapping signals, H-5, H-6), 3.83 (s, 3H, CO_2_**Me**), 3.71 – 3.61 (m, 3H, H-2’, H-5’, H-6’a), 3.60 (s, 3H, O**Me**), 3.45 (dd, *J* = 10.8, 6.0 Hz, 1H, H-6’b), 2.67 (dd, *J* = 12.9, 4.6 Hz, 1H, H-3a), 2.15 (s, 3H), 2.12 (s, 3H), 2.11 (s, 3H), 2.04 (s, 3H), 2.02 (s, 3H) all OAc, 1.94 (t, *J* = 12.5 Hz, 1H, H-3b), 1.88 (s, 3H, OAc); ^13^C NMR (126 MHz, cdcl_3_) δ 171.80, 170.82, 170.57, 170.31, 170.15, 169.92, 167.81 (all C=O), 103.91 (C-1’), 97.02 (C-2), 74.28 (C-3’), 73.02 (C-5‘), 72.47(C-6), 69.69 (C-2’), 68.55, 68.48 (C-4, C-4’), 67.75 (C-8), 66.72 (C-7), 62.29 (C-9), 60.54 (C-6’), 57.17 (O**Me**), 53.25 (CO_2_**Me**), 49.39 (C-5), 37.75 (C-3), 23.16, 21.31, 20.77, 20.75, 20.73, 20.68 (all Ac); ES-HRMS calcd for C_29_H_43_NO_19_Na 732.2327, found m/z 732.2320 [M+Na]^+^ .

**Siaα2,3-GalOMe**

Compound **S4** (0.12 g, 0.169 mmol) was treated with aqueous 1 M LiOH (5 mL) and the mixture was stirred for 16 h at rt. The reaction mixture was neutralised using Dowex H^+^ resin and filtered. After lyophilisation **S5** was obtained as a white solid (83 mg, quant.). The NMR data recorded for this compound is in good agreement with that reported in literature.^6^

^1^H NMR (500 MHz, d_2_o) δ 4.25 (d, *J* = 7.9 Hz, 1H, H-1’), 3.95 (dd, *J* = 9.8, 3.3 Hz, 1H, H-3’), 3.81 (dd, *J* = 3.2, 1.0 Hz, 1H, H-4’), 3.76 – 3.68 (m, 3H, overlapping signals, H-5, H-6, H-9a), 3.66 – 3.48 (m, 7H, overlapping signals, H-4, H-7, H-8, H-9b, H-5’,H-6’a, H-6’b) 3.43 (s, 3H), 3.40 (dd, *J* = 9.8, 7.9 Hz, 1H, H-2’), 2.62 (dd, *J* = 12.4, 4.7 Hz, 1H, H-3a), 1.89 (s, 3H, NHAc), 1.65 (t, *J* = 12.1 Hz, 1H, H-3b).

^13^C NMR (126 MHz, d_2_o) δ 174.97, 173.78 (both C=O), 103.46 (C-1’), 99.76 (C-2), 75.79 (C-3’), 74.86 (C-5’), 72.79 (C-8), 71.72 (C-6), 69.07 (C-2’), 68.27, 68.01 (C-4, C-7), 67.48 (C-4’), 62.50 (C-9), 60.91 (C-6’), 56.96 (O**Me**), 51.63 (C-5), 39.62 (C-3), 21.98 (NHAc).

ES-HRMS calcd for C_18_H_30_NO_14_ 484.1672, found m/z 484.1671 [M-H]^+^ .

**Compound S10**

Compounds **S6**^7^ (2.0 g, 4.2 mmol) and **S7**^7^ (3.68 g, 6.3 mmol) were dissolved in anhydrous CH_2_Cl_2_ and the solvent was removed and the residue dried under vaccuum for 3 h. A solution of SmI_2_ (0.1 M in THF, 7 equiv.) was transferred via cannula under positive pressure of N_2_ at room temp (colour change from dark blue to green observed over 5 min). The mixture was stirred for 15 min and then water added (yellow solution obtained), which was diluted with Et_2_O. The Et_2_O layer was washed with 1M HCl, aq sodium tartrate, dried (Na_2_SO_4_) and the solvent removed. Flash chromatography (toluene-acetone 7:3) yielded the C-disaccharides **S8** (R&S) and by-products. This mixture was dissolved in EtOAc and stirred with activated charcoal (20 g) for 16 h. Filtration through celite, and solvent removal gave **S8** (R&S, 2.72 g, 68%) as a white foam. This intermediate **S8** (2.2 g, 2.3 mmol) was dissolved in EtOAc and a mixture of 10% Pd-C and 20% Pd(OH)­_2_-C was charged and the flask was then subjected to 3 purge and refill cycles of hydrogen gas. The heterogeneous mixture was stirred under a balloon of H_2_ for 16 h. The flask was then purged with N_2_, followed by filtration through celite. The filtrate was condensed under reduced pressure. Flash chromatography (CH_2_Cl_2_-MeOH, 20:1 then 10:1) of the residue afforded **S9** (mixture of isomers, 1.34 g, 85%) as a white solid. Compounds **S9** (R&S, 0.58 g, 0.85 mmol) were suspended in dimethoxypropane (40 mL, 0.67 mmol) where CSA (20 mg, 0.086 mmol) was added, and the mixture stirred for 4 h. Et_3_N (0.02 mL) was added and the solvent removed under reduced pressure. Column chromatography (CH_2_Cl_2_-MeOH 20:1 then 10:1) of the residue gave **S10** (0.13 g, 20%); ^1^H NMR (500 MHz, cdcl_3_) δ 5.30 – 5.22 (m, 2H, overlapping signals. H-7, H-8), 5.12 (d, *J* = 10.2 Hz, 1H, NHAc), 4.77 (td, *J* = 12.8, 4.1 Hz, 1H, H-4), 4.40 (d, *J* = 7.3 Hz, 1H, H-1), 4.26 (m, 3H, overlapping signals, H-9a, H-6’a, CH(S)), 4.17 (d, *J* = 10.8 Hz, 1H, H-6), 4.08 – 3.93 (m, 4H, overlapping signals, H-5, H-9b, H-2’, H-4’), 3.83 (d, *J* = 12.6 Hz, 1H, H-6’b), 3.80 (s, 3H, CO_2_Me), 3.56 (s, 3H, OMe), 3.44 (s, 1H, H-5’), 2.44 (t, *J* = 12.8 Hz, 1H, H-3a), 2.20 – 2.13 (m, 7H, overlapping signals, H-3b, 2 x Ac), 2.09 – 2.01 (m, 7H, overlapping signals, H-3’, 2 x Ac), 1.91 (s, 3H, Ac), 1.48 (s, 3H, CC**H**_3_), 1.44 (s, 3H, CC**H**_3_), 1.36 (d, *J* = 2.3 Hz, 6H, 2 x CC**H**_3_); ^13^C NMR (126 MHz, cdcl_3_) δ 171.35, 171.14, 170.67, 170.22, 170.17, 170.15 (all C=O), 102.86 (C-1’), 99.38, 98.37 (both **C**CH_3_), 81.82 (C-2), 72.42 (C-5’), 70.24 (C-4), 69.91, 68.64, 67.85, 67.41, 67.29 (C-7, C-8, C-2’), 64.19 (C-4’), 62.90 (C-6’), 62.31(C-9), 56.32 (CO_2_Me), 52.45 (OMe), 49.65 (C-5), 41.27 (C-3’), 33.85 (C-3), 29.67, 29.64 (both CCH_3_), 23.27, 21.10, 20.96, 20.81, 20.77 (All Ac), 19.68, 19.30 (both CCH_3_); ES-HRMS calcd for C_34_H_51_NO_18_Na 784.3004, found m/z 784.3004 [M+Na]^+^ .

**Compound S11**

Compound **S10** (120 mg, 0.154 mmol) was dissolved in anhydrous MeOH (6 mL). To this solution was added p-TsOH and the reaction was stirred for 15 min. The reaction was monitored by TLC (1:1 toluene: acetone) and upon consumption of the starting material, was quenched using NEt_3_. The solvents were removed under reduced pressure and the residue was subjected to column chromatography (20:1 CH_2_Cl_2_: MeOH to 9:1 CH_2_Cl_2_: MeOH). The compound **S11** (0.09 g, 80%).

The NMR data recorded for **FB127** is in good agreement with that reported in a recent patent:^4^ ^1^H NMR (500 MHz, cdcl_3_) δ 5.28 (td, *J* = 7.5, 2.9 Hz, 1H, H-8), 5.22 (dd, *J* = 9.0, 1.8 Hz, 1H, H-7), 5.15 (d, *J* = 10.1 Hz, 1H, NHAc), 4.97 (td, *J* = 11.1, 4.5 Hz, 1H, H-4), 4.41 (d, *J* = 7.5 Hz, 1H, H-1’), 4.34 – 4.29 (m, 2H, overlapping signals, H-9a, CH(S)), 4.07 – 3.98 (m, 4H, overlapping signals, H-5, H-9b, H-2’, H-4’), 3.96 – 3.88 (m, 2H, overlapping signals, H-6, H-6’a), 3.84 (dd, *J* = 11.7, 5.6 Hz, 1H, H-6’b), 3.77 (s, 3H, CO_2_Me), 3.66 (t, *J* = 5.3 Hz, 1H, H-5’), 3.55 (s, 3H, OMe), 2.38 (dd, *J* = 13.5, 5.0 Hz, 1H, H-3a), 2.19 – 2.15 (m, 4H, overlapping signals, H-3b, Ac), 2.14 (s, 3H, Ac), 2.04 (dd, *J* = 2.3, 0.9 Hz, 6H, 2 x Ac), 1.91 – 1.84 (m, 4H, overlapping signals, H-3’, Ac), 1.46 (s, 3H), 1.40 (s, 3H) both Ac; ^13^C NMR (126 MHz, cdcl_3_) δ 171.47, 171.10, 170.92, 170.76, 170.25, 170.05 (all C=O), 103.32 (C-1’), 99.84 (**C**CH_3_), 82.17 (C-2), 76.62 (C-5’), 73.19 (C-6), 71.38 (C(S)), 70.02 (C-4), 68.49 (C-8), 68.07 (C-2’), 67.27 (C-7), 65.38 (C-4’), 62.77 (C-6’), 62.53 (C-9), 56.33 (OMe), 52.81 (CO_2_Me), 49.78 (C-5), 42.59 (C-3’), 32.07 (C-3), 29.59 (CCH_3_), 23.19, 21.17, 20.92, 20.77, 20.70 (All Ac), 19.73 (CCH_3_); ES-HRMS calcd for C_31_H_47_NO_18_Na 744.2691, found m/z 744.2676 [M+Na]^+^

**FB127 (S12)**

Compound **S11** (50 mg, 0.069 mmol) was treated with aqueous 1M LiOH (2 mL) and the mixture was stirred for 16 hours at room temperature. The reaction mixture was neutralised using Dowex H^+^ resin and was filtered. After lyophilisation the **S12** was obtained as a white solid (quant). The NMR data is in good agreement with that reported in literature.^7^ ^1^H NMR (500 MHz, d_2_o) δ 4.37 (d, *J* = 7.6 Hz, 1H, H-1’), 4.21 (d, *J* = 10.3 Hz, 1H, CH(S)), 4.11 (s, 1H, H-4’), 3.87 (dd, *J* = 10.9, 7.5 Hz, 1H, H-2’), 3.73 (dd, *J* = 11.8, 2.1 Hz, 1H, H-9a), 3.71 – 3.55 (m, 7H, overlapping singals, H-4, H-5, H-6, H-7, H-8, H6’a, H-6’b), 3.50 (dd, *J* = 11.8, 6.3 Hz, 1H, H-9b), 3.45 – 3.42 (m, 1H, H-5’), 3.41 (s, 3H, OMe), 2.30 (dd, *J* = 12.7, 2.0 Hz, 1H, H-3a), 1.91-1.86 (m, 4H, overlapping signals, H-3’, Ac), 1.77 (t, *J* = 11.3 Hz, 1H, H-3b), 1.38 (s, 3H, CCH_3_), 1.29 (s, 3H, CCH_3_); ^13^C NMR (126 MHz, d_2_o) δ 177.25, 174.87 (both C=O), 102.78 (C-1’), 100.75 (CCH_3_), 82.80 (C-2), 78.52, 73.48, 71.97, 71.92, 68.73 (C-4, C-5, C-6, C-7, C-8), 68.34, 68.18 (C-2’, C-5’), 64.35 (C-4’), 62.58 (C-9), 61.02 (C-6’), 56.32 (OMe), 52.11 (C-5), 42.41 (C-3’), 34.82 (C-3), 28.28 (CCH_3_), 21.95 (Ac), 18.92 (CCH_3_); ES-HRMS calcd for C_22_H_36_NO_14_ 538.2136, found m/z 538.2137 [M-H]^-^ .

**References:**

(1) F.X. Schmid, *Encyclopedia of Life Sciences*; John Wiley & Sons, Ltd, Ed.; John Wiley & Sons, Ltd: Chichester, UK, 2001; p a0003142.

(2) J. M. Antosiewicz, D. Shugar, *Biophys. Rev.* 2016, **8**, 163–177.

(3) J. Shi, T. Yang, S. Kataoka, Y. Zhang, A. J. Diaz, P. S. Cremer, *J. Am. Chem. Soc.* 2007, **129**, 5954–5961.

(4) Li, C. Yun; Liu, G. Jian; Du, W.; Zhang, Y.; Xing, G. Wen. A Novel O-Fucosylation Strategy Preactivated by (p-Tol)_2_SO/Tf_2_O and Its Application for the Synthesis of Lewis Blood Group Antigen Lewis^a^. *Tetrahedron Letters*2017,**58,**2109–2112.

(5) Wolf, S.; Warnecke, S.; Ehrit, J.; Freiberger, F.; Gerardy-Schahn, R.; Meier, C. Chemical Synthesis and Enzymatic Testing of CMP-Sialic Acid Derivatives. *ChemBioChem* 2012, **13**, 2605–2615.

(6) Dhakal, B.; Buda, S.; Crich, D. Stereoselective Synthesis of 5-Epi-α-Sialosides Related to the Pseudaminic Acid Glycosides. Reassessment of the Stereoselectivity of the 5-Azido-5-Deacetamidosialyl Thioglycosides and Use of Triflate as Nucleophile in the Zbiral Deamination of Sialic Acids. *Journal of Organic Chemistry* 2016, **81**, 10617–10630.

(7) Woods, R. J.; Murphy, P. V.; Yang, L.; Smith, H. M. K.; Hendel, J. Glycomimetics to Inhibit Pathogen-Host Interactions, US Patent 9,605,014, 2017.
